# Evidence-based uncertainty: do implant-related properties of titanium reduce the susceptibility to perioperative infections in clinical fracture management? A systematic review

**DOI:** 10.1007/s15010-021-01583-z

**Published:** 2021-02-13

**Authors:** Michael C. Tanner, Christian Fischer, Gerhard Schmidmaier, Patrick Haubruck

**Affiliations:** HTRG–Heidelberg Trauma Research Group, Center for Orthopedics, Trauma Surgery and Spinal Cord Injury, Trauma and Reconstructive Surgery, Heidelberg University Hospital, 69118 Heidelberg, Germany

**Keywords:** Stainless steel implants, Titanium implants, Infection, Trauma surgery, Prevention of infection, Implant related infection

## Abstract

**Background:**

Implant-associated infections (IAI) remain a challenging complication in osteosynthesis. There is no consensus or clear evidence whether titanium offers a relevant clinical benefit over stainless steel.

**Purpose:**

In this systematic review, we sought to determine whether the implant properties of titanium reduce the susceptibility to IAI compared to stainless steel in fracture management.

**Methods:**

A systematic literature search in German and English was performed using specific search terms and limits. Studies published between 1995 and 1st June 2020 in the Cochrane library, MEDLINE and Web of Science databases were included. Only clinical studies comparing titanium and stainless steel implants regarding the susceptibility to infections were selected for detailed review.

**Results:**

Five studies out of 384 papers were identified and reviewed. From the studies meeting inclusion criteria one study was a systematic review, two studies were randomized controlled studies (RCT) and two studies were of retrospective comparative nature of level IV evidence.

**Conclusion:**

Our results show that currently, no proven advantage for titanium implants in respect to IAI can be seen in contemporary literature. Implants preserving periosteal blood-flow and minimising soft-tissue trauma show statistically significant benefits in reducing the incidence of IAI. Clinical studies providing reliable evidence regarding the influence of titanium implants on IAI and investigating the susceptibility of titanium to infection are necessary

## Introduction

Implant-associated infections are a feared complication in the context of osteosynthesis which can lead from impaired union to amputation of affected extremities [1]. In addition they have an immense socio-economic impact and may prolong the successful treatment of patients considerably. Despite improvements in implants, perioperative antibiotic prophylaxis and intraoperative management, IAI remain a therapeutically challenge in trauma and reconstructive surgery.

Titanium’s supposed better biocompatibility raised aspirations for advantages towards prevention of IAI. Uncoated implants made of surgical steel, titanium or its many alloys (Titanium 6% Aluminum 7% Niobium – TAN) are currently the most commonly used implant types in Trauma surgery [2]. One marked difference of these metals pertaining to IAI is their surfacing, which has a direct impact on their biocompatibility. Stainless steel is usually polished electrically, therefore showing a very smooth surface, whereas titanium is microporous. This pitted surface leads to osseous integration, whereas stainless steel is surrounded by soft tissue, leading to an avascular space between the two surfaces that are filled with fluid [3]. Initial *in-vitro* and *in-vivo* studies directly comparing the two different types of implant materials showed a reduced susceptibility for titanium towards IAI in comparison with steel [2].

Almost three decades [4] after the introduction of titanium implants there is still no consensus or clear evidence whether titanium offers a relevant clinical benefit over stainless steel in the prevention of IAI. Hence, in this systematic review, we sought to determine whether the implant properties of titanium reduce the susceptibility to IAI compared to stainless steel in fracture management.

## Materials and methods

A systematic literature search was performed according to PRISMA guidelines. In particular, the Cochrane library, MEDLINE, and Web of Science databases were searched on February 12th and 19th of 2019 and again 1st of June 2020. Strict eligibility criteria (Table 1) were applied based on the PICO criteria utilized for this review. Only studies published between 1995 and 1st of June 2020 were included in the review. The strategies for the performed searches are shown in Tables 2, 3 and 4. The identified studies were scrutinized using the Critical Appraisal Skills Program (CASP) checklist [5].

**Table 1:** Summary of the eligibility criteria

Inclusion criteria	Exclusion criteria
English language	Duplicate studies
German language	Studies investigating in only infectious behavior of titanium or stainless steel implants
Clinical studies	Studies investigating in implants used for elective spinal and orthopaedic surgery
Titanium versus stainless steel implants in fracture treatment comparative studies	Studies investigating in implants used for endoprosthesis
Susceptibility of implant material to infections clearly discussed	Pathological fractures
	Studies investigating in implants used for trauma management in facial fractures

**Table 2:** Search strategy in the Cochrane library

Terms	Results
MeSH descriptor: [Infection] explode all trees	20,325
MeSH descriptor: [Titanium] explode all trees	748
MeSH descriptor: [Stainless Steel] explode all trees	265
MeSH descriptor: [Postoperative Complications] explode all trees	33,257
MeSH descriptor: [Inflammation] explode all trees	7505
#4 and #2 and #3	6
(#1 or #5) and #2 and #3	2
#6 OR #7*1995-current	6

**Table 3:** Search strategy in the Medline database

Terms	Results
1 Stainless Steel/	6775
2 "stainless steel".ti,ab,af	16,166
3 1 OR 2	16,166
4 titanium.ti,ab,af	48,065
5 Titanium/	31,363
6 4 OR 5	48,065
7 3 AND 6	2325
8 Infection/	35,800
9 "infection".ti,ab,af	1,085,492
10 infection/ OR postoperative complication/ OR bacterial infection/ OR implant associated infection/ OR inflammation/	529,409
11 8 or 9 or 10	1,518,218
12 7 and 11	167
13 limit 12 to yr = "1995 -Current"	146

**Table 4:** Search strategy in the Web of Science® database

Terms	Results
1 TI = "stainless steel*"	20,320
Indexes = SCI-EXPANDED, SSCI Timespan = 1995–2017	
2 TS = "stainless steel*"	64,642
Indexes = SCI-EXPANDED, SSCI Timespan = 1995–2017	
3 #1 OR #2 Indexes = SCI-EXPANDED, SSCI Timespan = 1995–2017	64,642
4 TI = "titanium*" Indexes = SCI-EXPANDED, SSCI Timespan = 1995–2017	43,030
5 TS = "titanium*" Indexes = SCI-EXPANDED, SSCI Timespan = 1995–2017	150,133
6 #4 or #5 Indexes = SCI-EXPANDED, SSCI Timespan = 1995–2017	150,133
7 #3 and #6 Indexes = SCI-EXPANDED, SSCI Timespan = 1995–2017	5864
8 TI = (infection* or "postoperative complication*" or "bacterial infection*" or "implant associated infection*" or inflammation*) Indexes = SCI-EXPANDED, SSCI Timespan = 1995–2017	331,482
9 TS = (infection* or "postoperative complication*" or "bacterial infection*" or "implant associated infection*" or inflammation*) Indexes = SCI-EXPANDED, SSCI Timespan = 1995–2017	1,370,554
10 TI = infection* Indexes = SCI-EXPANDED, SSCI Timespan = 1995–2017	260,052
11 TS = infection* Indexes = SCI-EXPANDED, SSCI Timespan = 1995–2017	1,016,147
12 #8 OR #9 OR #10 OR #11 Indexes = SCI-EXPANDED, SSCI Timespan = 1995–2017	1,370,554
13 #7 and #12 Indexes = SCI-EXPANDED, SSCI Timespan = 1995–2017	232

### Eligibility criteria

#### Participants

Patients suffering from a fracture of a bone of an extremity were included in the study regardless of age. Fractures of the vertebrae, skull, and mandibula, pathological fractures, and elective orthopaedic surgeries were excluded. A summary of the in- and exclusion criteria is demonstrated in Table 1.

#### Intervention

Surgical fracture fixation with titanium implants.

#### Comparator

Surgical fracture fixation with stainless steel implants.

#### Outcomes

1. Postoperative and perioperative infection of the implant and surgical site.
2. Susceptibility to perioperative infection associated with implant properties.

## Results

**Fig. 1:**
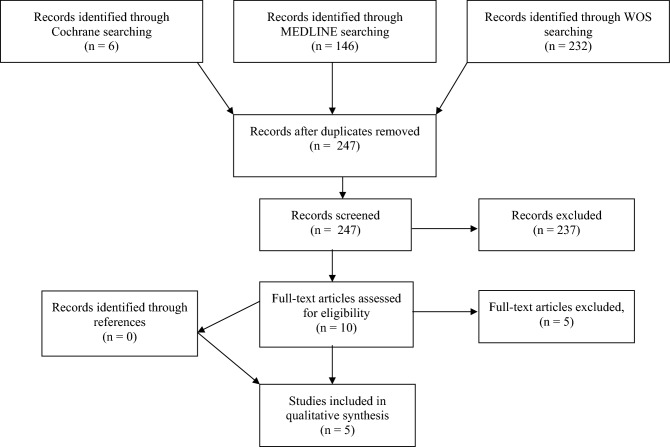
Flowchart of the record identification and selection process. Based on the PRISMA guidelines

A total of 384 studies were identified from the literature search (6 Cochrane library (Table 2), 146 MEDLINE (Table 3), 232 Web of Science (Table 4)). All duplicates were removed and all remaining titles and abstracts of the identified studies were checked for relevance by two independent reviewers (PH and MCT) according to the PRISMA guidelines. Finally, 10 studies were selected for full-text review following examination of their abstracts. Of these, 5 studies met the inclusion criteria. Additionally, all references of the articles selected for full-text review were checked for relevance according to the inclusion criteria (Fig. 1). All studies are summarized in Table 5.

**Table 5:** Display of the summary of results

Reference	Year of publication	Type of study	Title	Influence on implant related properties on the susceptibility to infection
Studies				
Mohamed et al	[5]	Systematic review	Clinical outcomes and complications of titanium versus stainless steel elastic nail in management of paediatric femoral fractures—a systematic review	No statistical difference
Pieske et al	[6]	RCT	Titanium Alloy Pins Versus Stainless Steel Pins in External Fixation at the Wrist: A Randomized Prospective Study	No statistical difference
Rozental et al	[8]	Retrospective clinical trial	Functional Outcome and Complications Following Two Types of Dorsal Plating for Unstable Fractures of the Distal Part of the Radius	No statistical difference
Voggenreiter et al	[9]	Retrospective clinical trial	Immuno-inflammatory tissue reaction to stainless-steel and titanium platesusedforinternalfixationoflongbones	No statistical difference
Arens et al	[7]	RCT	Infection after open reduction and internal fixation with dynamic compression plates—Clinical and experimental data	No statistical difference

From the studies that met the inclusion criteria one study was a systematic review, two studies were randomized controlled studies (RCT) and two studies were retrospective comparative studies of level IV evidence.

### Primary outcome

Mohamed et al. published a systematic review 2016 [5]. Here they compared the clinical outcomes and complications of titanium versus stainless steel elastic nails in the management of paediatric femoral fractures. A total of five studies matched the inclusion criteria (a total of 198 titanium implants and 183 stainless steel implants were compared) and were evaluated regarding infections after treatment with steel and titanium elastic nails. None of the included studies showed a significant difference in the rate of infection between the two groups [5]. A total of two RCT were identified using the mentioned literature research in our study. Pieske et al. [6] published an RCT in 2008 investigating titanium alloy pins versus stainless steel pins in external fixation of the wrist. A total of 80 patients (320 pins) were treated with external fixation of the wrist, in particular, 40 patients were treated with stainless steel implants and 40 patients with titanium implants. The data of the study revealed no statistical differences among the two groups with regard to the prevalence of pin-site complications. However, the rate of premature removal of fixator because of severe pin-track infection (5% vs. 0%) was higher in the stainless steel group [6]. Another RCT was published 1996 by Arens et al. [7]. The authors investigated the rates of infection after open reduction and internal fixation with dynamic compression plates using both titanium and stainless steel implants. A total of 281 cases were evaluated (154 patients were treated with stainless steel implants and 127 with titanium implants). Regarding IAI the authors stated that no statistical difference was observed between the two groups.

A total of two retrospective trials were identified during our literature research. Rozental et al. [8] published the results of a retrospective trial in 2003. A total of 28 patients that were treated with dorsal plating for unstable fractures of the distal part of the radius were analyzed (14 patients were treated with titanium implants and 14 patients with stainless steel implants). No patients showed an early or late infection, regardless of implant material, during the study period averaging 21 months [8]. Voggenreiter et al. [9] published a retrospective trial in 2003 investigating the immuno-inflammatory tissue reaction to stainless-steel and titanium plates used for internal fixation of long bones. The immuno-inflammatory response to a total of 43 implants (21 stainless steel implants and 22 titanium implants) was analyzed. The results of this trial revealed a marked immuno-inflammatory reaction in the tissue surrounding the implants regardless of the implant material [9].

### Secondary outcome

A total of three studies analyzed the influence of the implant properties on the susceptibility to IAI. Voggenreiter et al. stated that particles of both materials are phagocytosed by macrophages, which are activated and maintain chronic inflammation. No differences between the two materials with respect to this were detected [9]. The authors concluded that titanium was seemingly not as biologically inert as it was supposed to be several years before [9]. Pieske et al. mentioned that despite no statistical differences being detected, a higher incidence of severe pin-track infection occurred in the stainless steel group thereby supporting previous results indicating that titanium reduces the susceptibility to local infection [6]. Furthermore, Arens et al. [7] classified evaluated infection rates according to initial bacterial contamination after osteosynthesis with stainless steel and titanium implants and published the results in 1996. Regarding the susceptibility to IAI, their results showed that initial bacterial contamination and stainless steel implants tended towards a higher rate of IAI compared to titanium implants. However, the results were to a non-significant extent [7].

## Discussion

Peri- and postoperative implant-associated infections (IAI) pose a severe complication of osteosynthesis in trauma surgery, which can lead to delayed- or non-union and even amputation of the affected extremity. IAI are potentially disastrous complications that have a high socio-economic impact as they profoundly delay patient’s treatments. IAI may appear directly after surgery or in the extended postoperative course. Reasons for this are multifactorial and may be subdivided into the following risk-factors [10]:

1. Pathogenetic factors (i.e. fracture mechanism, location, initial contamination).
2. Patient individual factors (i.e. diabetes, immunosuppression, nicotine abuse, haematogenous contamination).
3. Iatrogenic factors (i.e. intraoperative contamination, unsterile instruments, perioperative contamination by the surgeon or other staff).
4. Implant associated factors (i.e. implant properties and condition, type of implant used).

It is common knowledge that closed fractures have a lower rate of infection than open ones (1,5% vs, 3–40%), especially considering that 60% of open fractures are initially contaminated by bacteria [11]. The incidence of IAI varies in the literature from 1% after surgical treatment of low-energy traumata up to 30% in complex open fractures [11, 12]. The past decades have shown a continuous decline in IAI, for reasons yet unknown [13]. One reason might be a more consequent soft tissue management and perioperative antibiotic therapy.

IAI are in most cases due to contamination secondary either to initial trauma or intraoperative contamination, for example with skin flora. All known bacteria or fungi are principally capable of inducing IAI. The most common culprits are coagulase-negative staphylococci (30–43%), staphylococcus aureus (12–23%), streptococci (9–10%), enterococci (3–7%) or gram-negative bacilli (3–6%) [14, 15].

Strategies and guidelines have been implemented over the last years to reduce the risk for peri-operative infections and here the implant material represents only one factor amongst many. In orthopaedic surgery, hematogenous infection plays a minor role representing 0.3–7% of IAIs [16] while the main source of contamination remains the patient’s skin and airborne particles from the theater personal [17]. Consequently, a study by Knobben et al. showed that behavioral changes of the theater personal and limiting not only the number of personal in the theater but also their movement led to a significant decrease of IAI [17]. Although it is commonly believed that the laminar airflow aids towards a reduction of IAI [17] evidence exists indicating that laminar airflow showed no benefit and was associated with an even higher risk for severe IAI after joint replacement [18] causing uncertainty. While these measures are intended to reduce the initial contamination of the surgical wound and implant itself, local and systemic antibiotic treatment are crucial to prevent the onset of IAI [19]. Here, novel strategies that have been given FDA approval include antibiotic coated intramedullary nails [20] and DAC coated intramedullary nails and plates [21, 22].

The major challenge in IAI is the formation of biofilms, a biologically active matrix of cells and extracellular substances that associate with a metallic surface [10]. Biofilms are composed of bacteria adhering to a foreign body protected by an organic polymer. This protective substance is composed of extra-cellular polysaccharides that constitute an insoluble slimy secretion produced by bacteria. It protects bacteria from antibiotics, disinfectants, and defense mechanisms of the host [10]. The development of biofilms is divided into 5 steps, beginning with the attachment of a micro-colony on a foreign body, maturation, and development of a stable biofilm that comprehensively protects the bacteria, up to dispersion of individual microorganism and their dissemination in the bloodstream [10]. Infection with a bacterial biofilm can lead to local interruption of osteointegration and dissemination of pathogens may cause sepsis with impeding multi-organ failure and ultimately death of the affected patient. A relevant factor in biofilm development is the implant’s composition. Numerous properties of implants have different effects on biocompatibility. It has been postulated that materials with increased biocompatibility might provide additional protection from biofilms [23]. However, it was shown that Staph aureus, Staph epidermidis and Pseudomonas aeruginosa are all capable of inducing biofilms on both steel and titanium implants [10].

Propionibacterium, recently reclassified as cutibacterium [24], are gram-positive, aerotolerant anaerobic bacteria of the normal skin microbiome [25]. While non-pathogenic colonization of cutibacterium can be found in the skin as well as the oral cavity and urogenital tract [26] recent studies identified Cutibacterium acnes as a causative pathogen of low-grade infection including generation of a biofilm only in the presence of an implant [27]. Diagnosis remains challenging as C. acnes is often considered no more than a contaminant of the bacteriological cultures [28] and treatment requires long-lasting antibiotic regiments and often surgical revision [28]. While the majority of IAI in context with C. acnes has been reported in shoulder arthroplasty [29] cases of C. acnes on osteosynthetic implant material have been identified [28] requiring implant removal and an average of 5.7 months of adjuvant antibiotic treatment [28]. Evidence regarding the influence of the implant material on IAI caused by C. acnes remains scarce. Interestingly, results from a recent animal study provided evidence that although C. acnes was able to generate biofilms on both titanium alloy and stainless steel a significantly denser biofilm was observed on titanium alloy [27]. The authors concluded that modification of the implant surface using nanotechnologies or coating strategies might prevent the onset of IAI caused by C. acnes [27].

Low-grade or late IAI are associated with less virulent bacteria such as C. acnes and S. epidermidis [30] and patients usually present with subtle or no clinical symptoms and elevated infectious laboratory parameters are missing. The most common sign of low-grade IAI is a compromised fracture healing and formation of a non-union [31]. Only a few studies have addressed the influence of implant material on low-grade IAI, here a recent study by Komnos et al. investigated the susceptibility of titanium alloy implants with different surface structures to low-grade IAI and found that modern osteointegrative surface topologies reduced the risk significantly [32]. While this strategy has important implications for joint replacing implants its transferability to osteosynthesis implant materials is limited as implant removal is common. Further studies are needed that investigate the influence of the implant properties on low-grade IAI.

Over 80% of microbial infections are due to biofilm generation [10]. Because of their numerous protective characteristics, biofilm contamination is extremely difficult to treat. Besides antibiotic treatment, most cases require surgical removal of the affected implant to successfully eradicate bacteria. This poses an additional risk for the patient. To protect them, it is sensible to use those implants which, due to their specific properties, help minimize the risk of biofilm formation. Currently, there are numerous types of implants with varying designs and properties for different anatomical regions and diverse osteosynthetic procedures. These properties and designs have a demonstrable effect on IAI.

Designs of current implants are continually being improved in terms of soft-tissue protection, periosteal preservation, and the guarantee of local microperfusion through the peri- and endosteum. Initial “dynamic compression plates” whose function was dependent on compression between plate and bone were soon superseded by the “limited contact dynamic compression plate” and “locking compression plate”, which further reduced contact of the implant with the periosteum. Reduction of soft-tissue injuries, improved stability and protection of the microvascular environment were all shown to aid in the reduction of IAI, regardless of implant composition [33]. Intact periosteal soft-tissues have a positive influence on local cellular and humoral response, thereby increasing immunogenic response in combination with local vascularization [33].

Despite the immense clinical impact of IAI in trauma surgery, there are only a few relevant publications, most of which only regard this problem in vitro or in animal studies. Seligson et al. [34] performed an animal study on sheep in which they compared the in-vivo behavior of titanium and steel implants, especially their susceptibility for infections. Results of the study showed a slight reduction of IAI for titanium, albeit without statistical significance [34]. In a comparable animal model Ganser et al. showed in 2007 [35] that in external fixators, there was no significant difference in the use of Schanz screws made either of titanium or steel towards IAI [35]. Two further studies conducted on rabbit models by Johansson in 1999 [36, 37] comparing conventional steel plates (S-DCP) with monocortical titanium plates (PC-Fix) showed no statistical difference in terms of IAI. The authors noted that foreign material in general increases susceptibility for infection. The authors were further able to show that, independent of their material, S-DCPs are more susceptible to haemogenous IAI [36, 37]. Hudetz et al. (2008) [38] and Harris et al. (2007) [39] performed in vitro studies examining the effects of implant materials on Staphylococcus-infections. They reached the conclusion that the materials used are merely an inferior factor in susceptibility and infection rate with Staphylococcus aureus [38, 39]. They did, however, take note that the electropolished titanium surface was less vulnerable to Staphylococci in-vitro [39]. An animal study recently published by Metsemakers et al. in 2016 [40] came to the conclusion that titanium implants have no effect on the rate of infection. Weckbach et al. published a clinical retrospective cohort study in 2012 [41] which concluded that using steel implants for selected fractures does not increase the revision or complication rates. Even though this study makes no direct comparison of materials used, results showed no increased incidence of IAI in comparison to other published results [41]. Metsemakers et al. published a review in 2016 [2] in which implant materials had no effect on outcome of operative treatment of fractures. They did, however, speculate that the design of implants, especially plates, might show influence on IAI [2]. Implants sparing periosteal perfusion and avoiding soft-tissue injuries are believed to show benefits in regards to IAI. The same results were seen in a review by Schlegel et al. in 2006 [23]: the authors took up the study by Arens et al. [7] and were able to show that the design of implants, especially the protection of periosteal perfusion, had an advantageous influence on the development of IAI.

Evidence in clinical studies regarding the influence of implant properties on IAI and the susceptibility to infection remain scarce. During our literature research, we were able to identify merely five studies investigating this relevant complication. Furthermore, only three studies addressed the susceptibility to infections in association with the implant properties. However, the results of this systematic review support previous findings gathered in in-vitro and in-vivo studies. The results published by Voggenreiter et al. in 2001 [9] supported Johansson`s results from 1999 [36, 37] and showed in a clinical environment that implants lead to a soft-tissue reaction and inflammation regardless of the material used [9]. A further prospective study by Pieske et al. from 2008 [6] showed a slight reduction of pin track infections of external fixators when titanium pins were used to bridge distal radius fractures, albeit without statistical significance. Arens et al. came to a similar conclusion in 1996 [7] in their study combining animal experiments and a prospective randomized study. This also showed a slight tendency towards reduction of IAI for titanium, again without noted statistical significance [7]. Contrary to this the animal experiment showed significant differences for titanium and steel DCP`s susceptibility to IAI with advantages for titanium over steel. These results were not supported by the clinical part of the study [7]. The results of Rozental et al. [8] showed that no patients showed an early or late infection during the study period averaging 21 months regardless of the implant material used. In dorsal plating of the distal radius, complications arose due to differences in the design of implants [8]. A systematic review by Mohamed et al. from 2016 [5] compared the influence of implant materials in elastic nailing of children’s femoral fractures on outcome and complications. The authors noted no difference in IAI dependent on implant materials [5].

## Conclusions

IAI are a clinically relevant challenge in the treatment of trauma patients. Evidence of clinical studies regarding the influence of implant properties on IAI and the susceptibility to infection remain scarce. Many treatment algorithms were adapted from joint replacement surgery since evidence-based conclusions for trauma patients are meager, if not non-existent [1]. The results of this review show that currently, no proven advantage for titanium implants in respect to IAI can be seen in contemporary literature. Solely slight tendencies towards a reduced susceptibility of titanium implants to IAI without statistical noteworthiness exist in the literature review. Despite this, implants that preserve periosteal blood-flow and reduce soft-tissue trauma have shown statistically significant benefits in reducing the incidence of IAI. Due to the results of the current review, we would like to strongly encourage further well-designed clinical studies to provide reliable evidence regarding the influence of titanium implants on IAI and investigate into the susceptibility of titanium to infection. Investigators planning studies in this field need to take the overall low incidence of IAI on osteosynthesis material into account when conducting a sample size calculation to ensure the adequate power of their studies.
